# Digital twin creation of a proton therapy treatment environment with hybrid LiDAR and RGB 3D camera

**DOI:** 10.1002/acm2.70231

**Published:** 2025-08-15

**Authors:** Jingjing M. Dougherty, Erik J. Tryggestad, Chris J. Beltran

**Affiliations:** ^1^ Department of Radiation Oncology Mayo Clinic Jacksonville Florida USA; ^2^ Department of Radiation Oncology Mayo Clinic Rochester Minnesota USA

**Keywords:** collision avoidance, LiDAR, proton therapy, 3D modeling

## Abstract

**Introduction:**

This study evaluates the feasibility of utilizing a commercial‐grade 3D LiDAR/RGB camera, combined with 3D reconstruction software, to create an accurate and hyper‐realistic digital model of a proton therapy treatment room. This reconstructed model aims to enhance collision avoidance strategies and improve 3D machine model accessibility. Utilization of a 3D LiDAR/RGB camera as a radiotherapy environment modeling has not been reported prior.

**Methods:**

To create a comprehensive 3D model of a half‐gantry proton therapy system, colored point cloud data was captured using the Matterport Pro 3 camera, a hybrid LiDAR and RGB imaging system with high‐resolution capabilities (134.2 MP), a 360° horizontal field of view, and 20 mm accuracy at 10 m. A total of 117 acquisition points, distributed across the treatment room with three camera heights, ensured complete coverage and minimal occlusions. Scanning, completed in under 3 hours, was monitored in real time using the Matterport App. Post‐scan processing involved denoising and converting the point cloud into 3D mesh structures using MeshLab, followed by refined pair‐wise iterative closest point (ICP) alignments. Textures and materials were assigned to reflect real‐world objects, and a ray‐tracing engine simulated realistic lighting in Blender. Animations illustrating the kinematics of the treatment couch and gantry were simulated in Blender for enhanced visualization.

**Results:**

The scanning process achieved complete data capture without missing information, attributed to strategic oversampling during data acquisition. The mean root mean square error (RMSE) of the ICP registration was 0.008 m. The validation process confirmed that the dimensions of the treatment couch within the digital model closely matched the actual measurements with less than 2 cm deviation, indicating high accuracy. The resulting digital twin provided a photorealistic and immersive representation of the proton therapy treatment room, serving as a valuable digital asset for various applications.

**Conclusion:**

It is possible to generate vendor‐independent and highly accurate 3D models of the proton treatment room environment with a commercial grade LiDAR/RGB camera to expand future research opportunities and education endeavors.

## 1 INTRODUCTION

The precision of proton therapy in cancer treatment relies on accurate and safe delivery techniques. A key component of this process is ensuring collision avoidance between the treatment equipment and the patient. In radiation therapy, collision detection solutions can be broadly categorized into two groups. The first includes vendor‐provided software modules, which range from basic treatment machine geometry modeling to more advanced 3D computer‐aided design (CAD) modeling, such as the Radformation (New York, NY) ClearCheck Collision Checks. The second category encompasses user‐developed, practical in‐house techniques. These solutions include mathematical approximations of collision‐free space,^1^, ^2^, ^3^, ^4^, ^5^ stand‐alone graphical simulation software,^6^, ^7^, ^8^, ^9^, ^10^ scripting APIs to create integrated 3D collision detection tools,^11^, ^12^, ^13^, ^14^, ^15^, ^16^ and the use of pre‐existing patient and machine surface models to predict collision‐free spaces.^17^, ^18^, ^19^, ^20^ Most of these collision avoidance solutions incorporate 3D CAD models of treatment equipment, typically provided by proton system vendors. This is because rendering these models is often proprietary, complex, and beyond the technical expertise of most end‐users to create accurate representations. However, using detailed 3D CAD models for collision simulation poses challenges, including limited availability of open‐source models from the vendors and variability in treatment room designs across different institutions. These differences make it difficult to adopt a standardized 3D CAD model that suits universal applications.

In this study, we present a pragmatic approach to generate highly accurate and hyper‐realistic treatment room models using a commercial grade 3D Light Detection and Ranging (LiDAR) camera and open‐source 3D reconstruction and rendering software. LiDAR sensors have revolutionized multiple fields by providing high‐resolution, three‐dimensional spatial data. In the realm of autonomous vehicles, LiDAR plays a pivotal role in real‐time mapping, obstacle detection, and navigation.^21^ Environmental monitoring also benefits greatly from this technology, as it aids in forest canopy analysis, flood risk assessment, and coastline mapping.^22^ Urban planning has seen significant advancements through LiDAR, enabling precise modeling of infrastructure and energy consumption.^23^ Furthermore, archaeological surveys have been transformed by LiDAR, uncovering hidden structures beneath dense vegetation.^24^ Additionally, indoor 3D modeling with LiDAR sensors serves a wide range of purposes, such as indoor construction planning,^25^ cultural heritage preservation^26^, ^27^ and providing foundational data for virtual reality applications in real estate and gaming.^28^ The cost of a short‐range LiDAR sensor has significantly decreased in recent years, making it a popular technique for practical indoor mapping.

By applying similar data acquisition and processing techniques from indoor scene mapping,^28^ we evaluated the feasibility of generating a viable 3D point cloud model of a proton therapy treatment room with a commercial LiDAR camera—Matterport Pro3. We then post‐processed the series of treatment room point cloud data to generate a single 3D dataset. The final 3D point cloud model is further transformed and refined into a hyper‐realistic digital twin of the treatment room environment with open‐source 3D rendering software MeshLab and Blender. Proton treatment couch and gantry movements were simulated using Blender to create an immersive user experience. The dimensions of selected equipment were validated in the 3D point cloud data to demonstrate the viability of this semi‐automated modeling framework.

## 2 METHODS

### 2.1 Theory

LiDAR sensors collect dimensional information in the form of point clouds, which are sets of data points in 3D space referenced in a Cartesian coordinate system (X, Y, and Z) in meters. In each data point, X represents the horizontal distance, Y represents the lateral distance, and Z represents the elevation or height, see Figure S1. Each point can sometimes also include additional attributes such as color, or reflectance. The reflectance attribute provides information about the surface characteristics, based on the reflectance of the laser pulse that hit the surface. Each point may also include a timestamp indicating when the data was captured. Timing information is useful for aligning data from multiple scans during post‐processing. To generate a robust 3D point cloud model without occlusions, repeated sampling at a wide range of locations is required for an indoor scene. After point cloud data collection, image registration using the camera's intrinsic parameters is necessary to create a comprehensive 3D model referenced in a unified coordinate system. Both spatial parameters (X, Y, Z) and the photometric data (RGB color) are considered as intrinsic parameters and can be utilized for point cloud image registration. There are four general steps involved in point cloud data registration: (1) preprocessing point cloud data to remove noise or reduce the number of points for faster processing, (2) initial alignment of the two point clouds into approximate alignment, (3) refine registration of the two point clouds with iterative closest point (ICP) algorithms,^29^ and (4) verification of the registration by computing the root mean square error (RMSE) between corresponding points, see Supporting Information for detail explanation of the RMSE definition. The most important step with impact to the final registration accuracy is the ICP algorithm selected, but in general, the algorithm minimizes a combined error function that includes both spatial and color distances between the corresponding points,^30^ p(target) and q(source):

$$\begin{array}{cc} & E\;\left(R,\;T\right)\\ & \;=\sum_{i}^{N}(w_{s}∥p_{i}^{xyz}-\left(Rq_{i}^{xyz}+T\right){∥}^{2}+w_{c}∥p_{i}^{color}-q_{i}^{color}){∥}^{2} \end{array}$$

where:

- $p_{i}^{xyz}$ and $q_{i}^{xyz}$: 3D coordinates of the corresponding points in the target and source point clouds.
- $p_{i}^{color}$ and $q_{i}^{color}$: Color attributes of the corresponding points in the target and source point clouds.
- R: 3 × 3 rotational matrix.
- T: 3 × 1 translational vector.
- $w_{s}$ and $w_{c}$: Weighting factors for the spatial and color distances, respectively.

For each point $q_{i}^{xyz}$ in the source point cloud, the algorithm iteratively finds the closest corresponding point in the target point cloud based on Euclidean distance. The total error E(R,T) of alignment is minimized between the two matched points with iteratively updated transformation (R,T) until the optimal rigid transformation is found. The weights $w_{s}$ and $w_{c}$ control the relative importance of the spatial and color parameters. For instance, in textureless regions, the geometric features are the dominant component, whereas in regions with strong color variation, the color features are the dominant component. Open‐source tools like MeshLab^31^ and Open3D^32^ can facilitate this registration process of multiple point cloud datasets, as well as the visualization of the point cloud data.

### 2.2 Matterport Pro 3

The Matterport Pro 3 is a cutting‐edge hybrid 3D camera designed to capture highly accurate digital twins of both indoor and outdoor spaces. This commercial system was selected due to its cost value and ease of operation. The hybrid system leverages advanced LiDAR technology to provide precise spatial data, while simultaneously capturing high‐resolution RGB images that are spatially aligned with the LiDAR camera field of view (FOV). The imaging sensors provide a resolution of up to 134.2 MP for a single panoramic image. The horizontal FOV is 360° and the vertical FOV is 295°. The wide FOV ensures comprehensive coverage of the scanned area per camera position. The depth sensing technology utilizes a Class 1 laser with a single wavelength of 904 nm. The accuracy of the LiDAR sensor is 20 mm at 10 m and a maximum range of 100 m. The camera can complete a single panoramic scan in less than 20 s. The camera only weighs 2.2 kg, making it extremely fast to setup. The operation of this system can be done wirelessly using the Matterport App on an Apple device, such as an iPad, for data pre‐viewing.

### 2.3 Point cloud data acquisition

**FIGURE 1:**
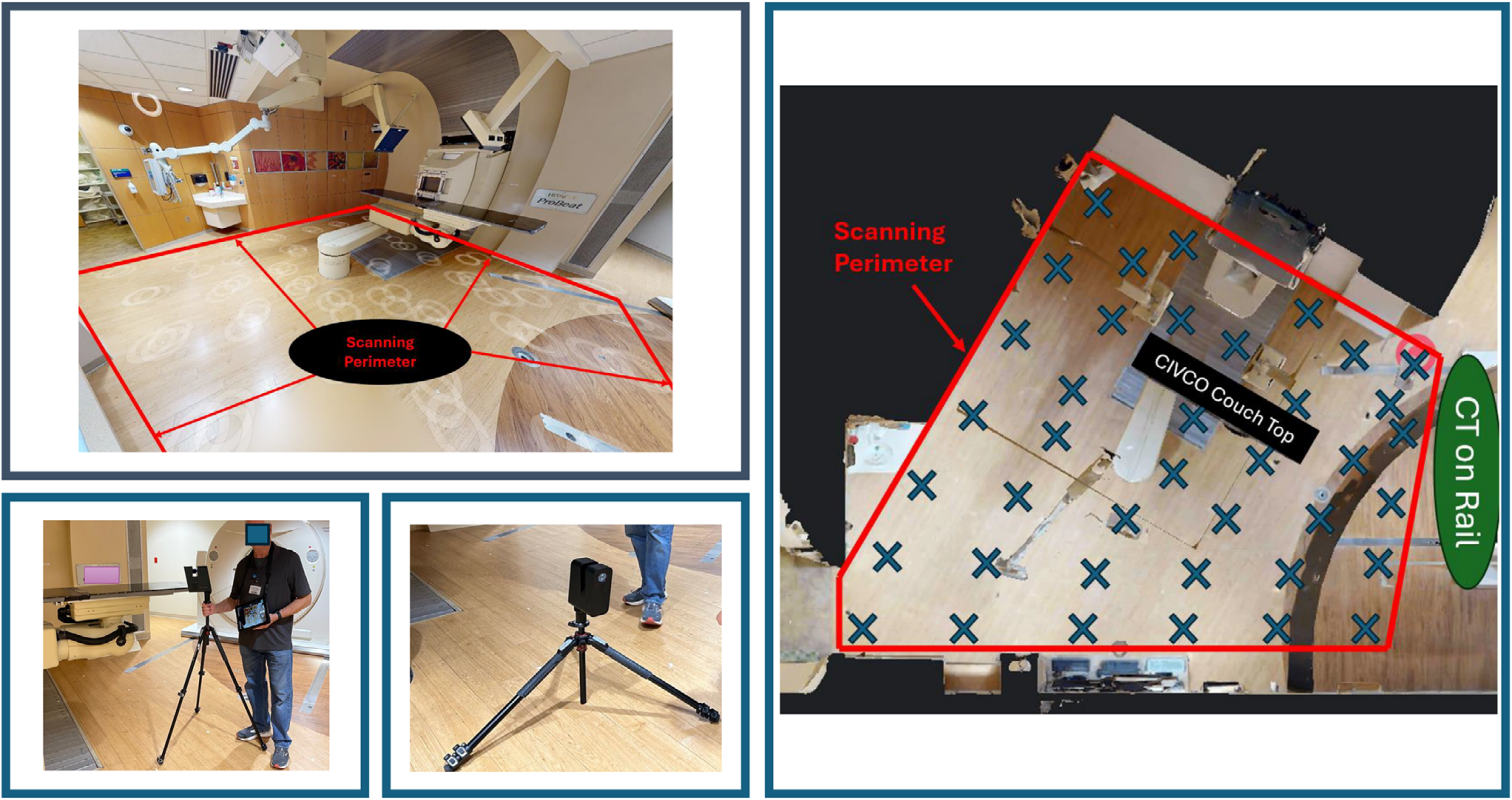
On the top left and the right of this figure, the red lines define the farthest boundaries where the Matterport camera was positioned. The “x” symbols indicate the planned camera acquisition locations. The treatment couch top is located approximately near the center of the scanning perimeter. The CT‐on‐Rail system is located outside of the scanning perimeter. The bottom of the figure shows two of the three camera acquisition heights: mid‐position and the low position.

A half‐gantry proton therapy system was chosen as the testing environment for the point cloud model reconstruction process. As shown in Figure 1, the total scanning area is 640 by 550 cm. The lighting condition of the treatment room was carefully adjusted by eliminating any halogen light source that could potentially interfere with the LiDAR laser. A total of 120 acquisition points were identified. These points are equally distributed throughout the treatment area with approximately 90 cm apart. Three camera heights were chosen as well to provide overlapping scanning data in the vertical direction. These overlapping scans ensure a comprehensive data collection of the objects inside of the treatment room from all viewing angles with minimal occlusion. The total scanning time was less than 3 h once the Matterport scanner was setup. Because the Matterport App provides real‐time image registration and viewing function, any erroneous or incomplete datasets were corrected immediately by re‐scanning and adjusting camera positions. Matterport App also provides a cloud‐based reconstruction service that will automatically generate a 3D point cloud of the scanning environment within 48 h. The final point cloud datasets were downloaded and exported for refined post‐processing and object dimension validation in MeshLab with an Apple M2 workstation (8‐core CPU and 10‐core GPU). The dimensions of the treatment couch and the robotic arm were measured using the MeshLab toolbox and compared to the actual dimensions of the objects.

### 2.4 Open‐source 3D creation suite‐blender

Blender^33^ is a versatile software that supports the entire 3D model creation pipeline, including modeling, rigging, animation, simulation, etc. The final reconstructed 3D point cloud data was denoised and transformed into 3D mesh structures in MeshLab. The mesh structures were then imported into Blender for further refinement. Based on the real‐world objects in the treatment room, materials and textures were assigned to mesh elements. The Cycles ray‐tracing rendering engine was added to simulate real‐world lighting behavior for highly realistic rendering of the treatment room. For kinematics simulations, motions of the treatment couch and proton gantry were simulated to give life to the hyper‐realistic model in Blender. For this Hitachi half gantry system, we demonstrated the capability of kinematics simulation within the Blenders animation workspace, a sequence of keyframes were added to the individual hardware components to create illustrations of movements of the gantry, robotic arm, and the treatment couch. As shown in Supporting Information, the animation shows the gantry rotating from 90° to 135°, the treatment couch rotates across a range of 190°, as well as the ability to simulate pitch, roll, and yaw correctional shifts of the robotic arm.

## 3 RESULTS

**FIGURE 2:**
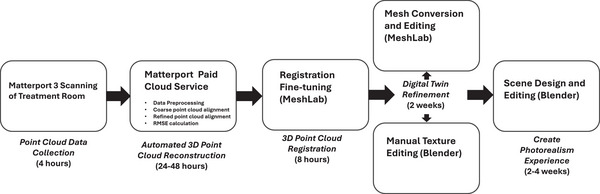
Final data acquisition and processing pipeline employed in this study with the estimated time took to complete each process.

The overall final data collection and processing pipeline is summarized in Figure 2. The total raw point cloud data acquisition was around 4 hours, including equipment setup and software connection. The automated Matterport data processing service provided a 3D point cloud data in 2 days’ time. The data inspection and fine registration were roughly around 8 hours. The mesh structure conversion, editing, and texturization took 2 weeks for objects deemed important for the collision avoidance simulation. The additional kinematic simulation process took another 3 weeks to complete. In total, this digital twin creation campaign took 6 weeks to complete from data collection to the application phase. All data were collected without issues and resulted in the successful mapping of the proton treatment room. The longest time was spent on the animation creation process, which is not necessary for the purpose of clinical implementation of a functional collision avoidance solution.

**FIGURE 3:**
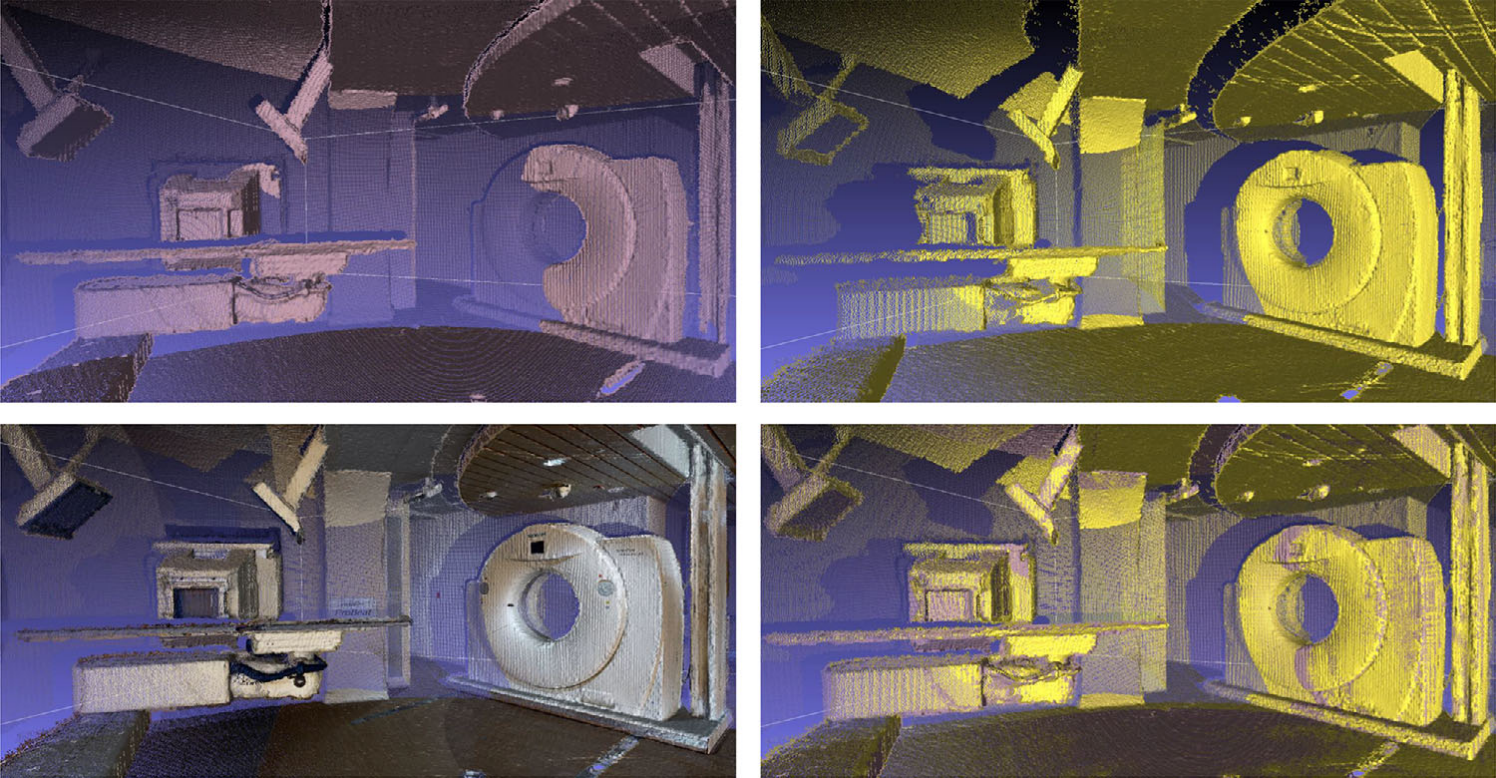
On the top right and left side of the figure are two separate scans out of the 117 datasets. The two scans are close in proximity with slightly different data collection field of view (FOV). The bottom right window shows the two scans overlaying with each other after a pairwise iterative closest point (ICP) registration with a root mean square error (RMSE) of 0.007 m. The bottom left window shows the combined color point cloud data after the ICP registration.

**FIGURE 4:**
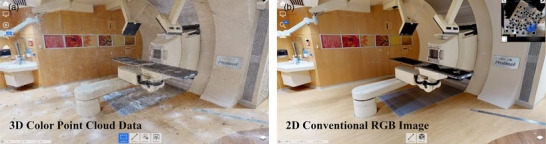
On the left side of the figure is an example image of the Matterport color 3D point cloud data of the half‐gantry proton therapy treatment environment. On the right side is the RGB image of the same field of view (FOV) as the point cloud acquisition. It is assumed that each image has the same camera position and viewing angle since the light detection and ranging (LiDAR) and RGB sensors were cross calibrated by the vendor.

After removing outlier points and denoising of the original point cloud data, roughly three million data points were preserved per acquisition position. An example of the ICP registration operation is shown in Figure 3. A total of 348 million points from 117 scanning points were registered together during post‐processing for further fine adjustments to increase scanning accuracy. The mean RMSE value was 0.008 m for the refined registration with the build‐in ICP algorithm in MeshLab. Figure 4 shows a partial FOV comparison between the LiDAR point cloud data and the high‐resolution RGB camera. Since the Matterport system is a commercial system, we were not able to evaluate its image calibration accuracy between the LiDAR point cloud image and the RGB image.

Table 1 presents the actual object dimensions, the mean and the standard deviation of the measured object dimensions in the final reconstructed 3D point cloud after ICP fine‐tuning, and the differences between the two values. The CIVCO couch top width, length, and height were selected for dimension verification. The CT‐on‐Rail scanner inner bore diameter was also compared. The mean differences between the actual and the measured dimensions were 0.6 cm (width), 1.8 cm (length), and 0.05 cm (height) for the couch top and −1.25 cm for the CT bore diameter.

**TABLE 1:** Dimension verification of the final 3D point cloud model using various reconstructed objects within the treatment room. The CIVCO couch top is located near the center of the scanning perimeter, and the CT‐on‐Rail scanner is located outside of the scanning perimeter, as shown in Figure 1.

Objects	Actual (cm)	Measured (cm)	Difference (cm)
CIVCO couch width	53	52.4 ± 0.15	0.6
CIVCO couch length	243.3	241.5 ± 0.35	1.8
CIVCO couch height	5	4.95 ± 0.085	0.05
CT bore diameter	80	81.25 ± 0.45	−1.25

**FIGURE 5:**
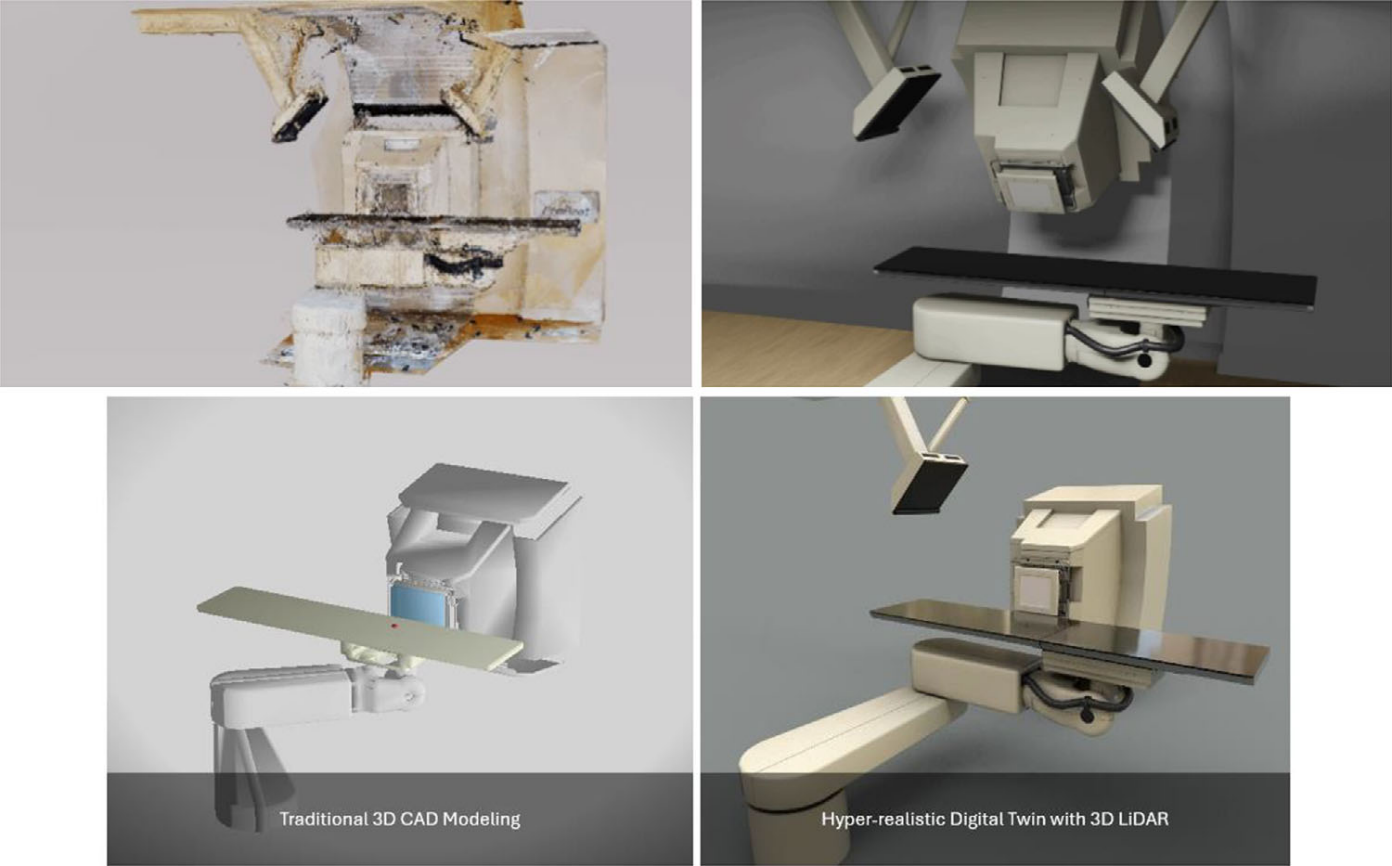
Top left window is an example segment of the final reconstructed 3D color point cloud; and the top right window is the final photorealistic model depiction of the rotating gantry and moving robotic couch. The bottom left window is the traditional rendering of the same treatment equipment with 3D computer‐aided design (CAD) modeling; and the bottom right window is the final digital twin model with physics‐based ray tracing lighting rendering.

As shown in Figure 5, the point cloud data was transformed into a programmable mesh structure during 3D model post‐processing, resulting in a hyper‐realistic representation of the proton treatment room. Key components critical for clinical collision avoidance scenarios—such as the proton treatment nozzle, treatment couch top, and robotic arms—were carefully refined to reflect their actual characteristics. Realistic materials and color schemes were applied to these objects, and a physically based ray‐tracing rendering engine was utilized to enhance the model's visual realism. Compared to traditional 3D CAD renderings of the same proton treatment room, the new digital twin offered a more immersive and authentic depiction, providing a superior visualization of the clinical environment.

## 4 DISCUSSION

The accuracy of the final 3D point cloud model has exceeded the expectations, considering the accuracy of the LiDAR equipment selected and its price point. During the point cloud collection, the Matterport Pro provides three levels of point cloud density that accommodate the need of different projects. The standard‐density captures about 800k points per scan, the medium‐density captures roughly 1.5 million points per scan, and the high‐density scan captures over five million points per scan. We chose the high‐density scanning mode for our experiment to enhance the ability of capturing high‐level details. The large number of scanning positions could also increase registration accuracy with the overlapping point clouds. Multiple scans from different angles increase the overlap between point clouds, facilitating better correspondence matching during ICP registration. We believe that the 2 cm accuracy is enough, depending on the individual institution's clinical workflow. As shown by a previous work,^11^ the collision avoidance solution has only an accuracy of 3 cm, and the clinical implementation in over a thousand patients proves the feasibility of our simulation software. The accuracy of the acquired LiDAR data is highly dependent upon the accuracy of the LiDAR hardware. In this study, the Matterport Pro 3 has a limited 2 cm range accuracy. However, with limited funding available, the Matterport Pro 3 system is what we have access to. More expensive systems are available with much higher accuracy in the millimeter range for other users to choose from if they have the financial means. Far more expensive 3D LiDAR scanners are available to achieve the same modeling task with even better accuracy (1–6 mm), but the price of these scanners is in the range of tens of thousands of dollars, making it challenging to justify the cost at this stage of our study. Based on the results from this initial study, we can reasonably hypothesize that the accuracy of the future models can only increase when more accurate LiDAR equipment is employed. High‐density point cloud scanning and large amounts of overlapping points are the keys to enhance ICP registration accuracy. The real‐time data visualization and registration function of the Matterport App also made it extremely easy to spot any acquisition errors or occlusions. Our refined registration process achieved an excellent RMSE value, which provided further confidence in our current digital twin creation process. We hope to further increase the modeling accuracy and provide a standardized process for digital twin creations of other radiotherapy treatment rooms as a future research direction.

Indoor scene mapping is not a new and novel technique, but to the best of our knowledge, the utilization of the LiDAR/RGB scanner as a radiotherapy environment modeling has not been reported prior. In comparison to other indoor mapping technologies, such as photogrammetry^34^ and the depth camera,^35^ LiDAR or laser mapping provides several advantages. The measurement accuracy with laser technology is far superior to the stereo‐sensing or photogrammetry methods. As demonstrated in the study, even with a commercial grade system, we can achieve high accuracy in object modeling. The LiDAR scanner can map objects even in extremely bright, dark, or low‐light conditions, since the sensor has its own laser emitting source. The range detection ability of a LiDAR sensor doesn't require daily calibration, but coordinate system calibration is still required when combined with other LiDAR sensors or sensors of a different modality. Conversely, the LiDAR scanner accuracy is affected by surfaces with low reflectivity, such as glass and dark surfaces. The ability of LiDAR to measure a distance relies on the signal strength of the reflected laser pulses, as more lights are absorbed or lost with dark surfaces or transparent surfaces. To combat these shortcomings, a light dusting powder can be applied to the dark or transparent surfaces. Alternatively, if a system allows, the gain of the laser strength can also be increased while imaging low reflectivity objects. This type of LiDAR system is known as the High Dynamic Range system, which is more expensive.

There are a few drawbacks of this study. First, we were not able to quantify the errors induced in the post‐processing of the point clouds, since the Matterport software is a proprietary product. The outlined data collection and processing steps are not completely automated, which will still require experienced users to manually edit the 3D mesh model. However, when compared to a manual 3D CAD model design from scratch, the LiDAR based 3D object mapping is far more efficient than the traditional 3D rendering efforts. We were not able to verify the LiDAR and RGB sensor cross‐calibration to ensure pixel position accuracy due to it being a commercial product. With any large‐scale dataset, the computational requirement is extremely demanding, requiring CPU multi‐core parallel processing, GPU acceleration, and large memory. It might not be feasible for smaller community hospitals to purchase their own hardware environment.

For the next stage of the project, we aim to provide detailed methods and results in patient external geometry modeling using LiDAR sensors. We will validate the 3D data reconstruction accuracy using an anthropomorphic phantom against its CT contours obtained from a treatment planning system. This extension of patient geometry simulation enables us to combine the LiDAR modeled machine geometry with the patient geometry for realistic treatment beam configuration simulation in a separate software platform, which is the goal of our future research works. Another foreseeable challenge is the range uncertainty associated with patients who have darker skin tones. Since darker surfaces absorb more light, less light is reflected back to the sensor for range estimation. The newer generation of LiDAR sensors can adjust their light output based on an object's color and reflectivity. We plan to conduct a detailed investigation into the accuracy of 3D surface reconstruction with diverse skin tone human subjects in the near future.

To support future applications involving patient‐specific simulations, a natural extension of this work involves the integration of independently acquired patient or phantom geometries with the digital twin of the treatment room. Since such surface reconstructions are typically captured using a different LiDAR sensor system optimized for fine‐detail 3D acquisition, merging them with the room model requires accurate geometric alignment. Potential methods include rigid registration based on fiducial markers or couch top alignment, coordinate transformations using known spatial references, or surface plane matching algorithms.^36^, ^37^ More advanced approaches, such as pose graph optimization, visual SLAM,^38^ or learning‐based point cloud registration (e.g., Deep Closest Point,^39^ PointNetLK^40^), may provide robust alignment in complex clinical settings. These techniques will be critical to enabling full‐scene simulation of treatment delivery scenarios involving both patient and hardware geometry.

Traditionally, 3D CAD renderings provided by vendors have been the gold standard for modeling hardware equipment used in radiotherapy collision avoidance. However, these models require user validation to ensure accuracy before integration into collision avoidance software. Our study introduces a vendor‐independent framework for generating precise equipment models for any radiotherapy system. This approach not only eliminates reliance on vendor‐provided models but also promotes accessibility by making these models available in open‐source formats for scientific research and kinematic modeling. This is critical for simulating equipment positions and interactions within the treatment environment. Moreover, the photorealistic representation of the proton treatment room bridges the gap between the clinical environment and users, enabling effective visualization and troubleshooting of potential collision issues before they impact patient safety. From an educational perspective, these highly realistic models provide immersive training tools for radiation oncology trainees, enhancing their understanding, engagement, and proficiency in managing collision avoidance processes.

## 5 CONCLUSION

This study demonstrates the feasibility of generating a vendor‐agnostic and highly accurate digital twin model of a proton therapy treatment room using a commercial grade 3D LiDAR/RGB camera. The resulting model offers significant potential for enhancing collision avoidance strategies and efficiency in radiotherapy treatment planning.

## AUTHOR CONTRIBUTIONS

JMD conceived and planned the studies. JMD, EJT, and CJB contributed to the interpretation of the results. JMD took the lead in writing the manuscript. All authors provided critical feedback and helped shape the research, analysis, and manuscript.

## CONFLICT OF INTEREST STATEMENT

The authors declare no conflicts of interest.

## Supporting information



Supporting information

Supporting information

## Data Availability

The data that support the findings of this study are available from the corresponding author upon reasonable request.
