# Supplementary material for: Digital twin creation of a proton therapy treatment environment with hybrid LiDAR and RGB 3D camera
Source: J Appl Clin Med Phys. 2025 Aug 15;26(8):e70231. doi: 10.1002/acm2.70231 (PMC12356692; doi:10.1002/acm2.70231)
Supplement: Supplementary file 1 — Supporting information [file ACM2-26-e70231-s002.docx]

# **Supplemental A**

Definition of RMSE:

The root mean square error (RMSE) in Iterative Closest Point (ICP) registration quantifies how well two point clouds data sets align after applying an estimated transformation. For each point in the source point cloud, the closest corresponding point in the target point cloud is identified. After applying the rotational and translational transformation to the source point cloud, the Euclidean distance between each transformed source point and its corresponding target point is computed. The distance values are summed, squared over all available data points, and then divided by the number of the total data points. A low RMSE value indicate a better alignment between the source and the target point cloud data, and it is being used as a metric to evaluate when the ICP algorithm has converged. The formula can be expressed as:

$$RMSE=\sqrt{\frac{1}{n}\sum_{i=1}^{n} \left\| T(P_{i})-Q_{i} \right\|^{2}}$$

Where:

$n$ is the total number of point clouds,

$P_{i}$ is the source point cloud,

$Q_{i}$ is the corresponding target point cloud,

$T$ is the estimated transformation matrix by the ICP algorithm.


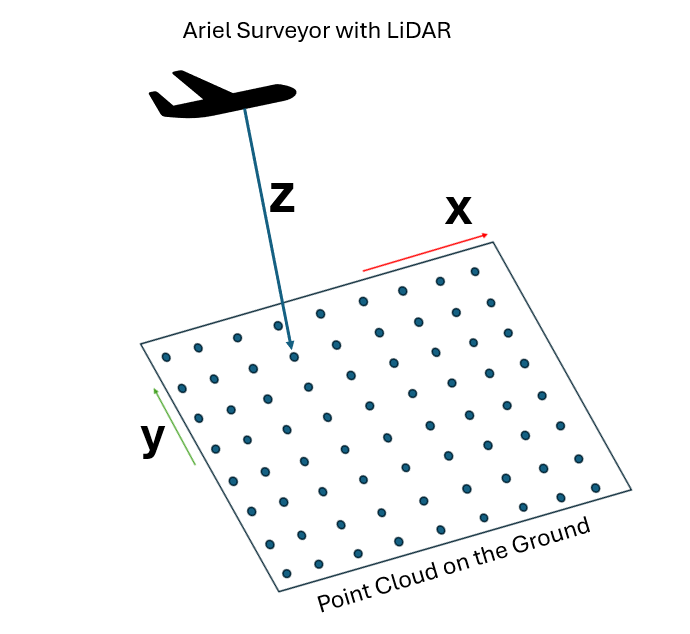


Figure S1: Illustration of a set of point cloud data point in 3D space with respect to the LiDAR camera on the plane surveying the earth. X represents the horizontal distance, Y represents the lateral distance, and Z represents the height or the elevation respect to the ground.
